# Retrograde Jejuno-jejunal Intussusception after Roux-en-Y Gastric Bypass: A Potential à la Mode Complication

**DOI:** 10.7759/cureus.3045

**Published:** 2018-07-24

**Authors:** Fady G. Haddad, Vera Zaraket, Iskandar Barakat, Liliane Deeb

**Affiliations:** 1 Gastroenterology and Hepatology, Staten Island University Hospital, New York, USA; 2 Gastroenterology, Staten Island University Hospital, New York, USA

**Keywords:** intussusception, bariatric surgery, roux-en-y, gastric bypass

## Abstract

Intussusception is a form of bowel obstruction caused by telescoping of a bowel segment into the adjacent part. Small bowel (SB) intussusception was previously considered a rare long-term complication of Roux-en-Y gastric bypass surgery (REYGB). However, with the rapid increase in the number of bariatric surgeries, the incidence of SB intussusception has been significantly increasing. This condition is potentially life-threatening if not recognized in a timely fashion. Herein, we report a case of retrograde jejuno-jejunal intussusception occurring six years after REYGB.

## Introduction

Intussusception, a rare event in adults, is a form of bowel obstruction caused by telescoping of a bowel segment into the adjacent part. Small bowel (SB) intussusception, once believed to be an uncommon long-term complication of Roux-en-Y gastric bypass surgery (REYGB) is becoming more prevalent with the rapid rise in the bariatric surgery rate over the past 20 years. This condition is potentially devastating if not promptly diagnosed and its etiology remains unclear. Herein, we report a rare case of retrograde jejuno-jejunal anastomotic intussusception that occurred six years after REYGB.

## Case presentation

**Figure 1:**
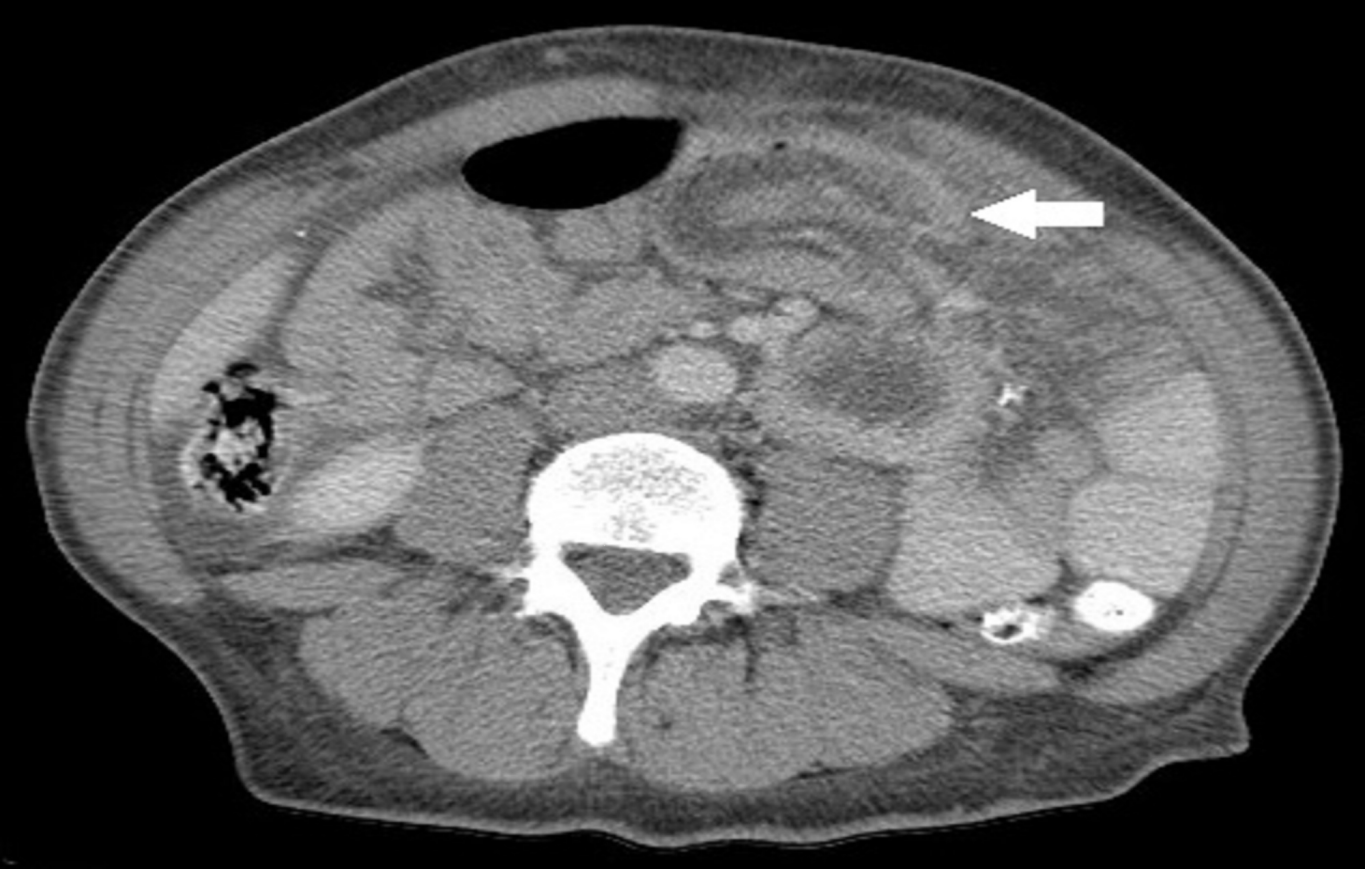
Abdominal computed tomography (CT) scan, axial view, showing dilatation of the biliopancreatic limb of the REYGB with SB intussusception (arrow) through the jejunojejunostomy REYGB: Roux-en-Y gastric bypass surgery; SB: small bowel.

**Figure 2:**
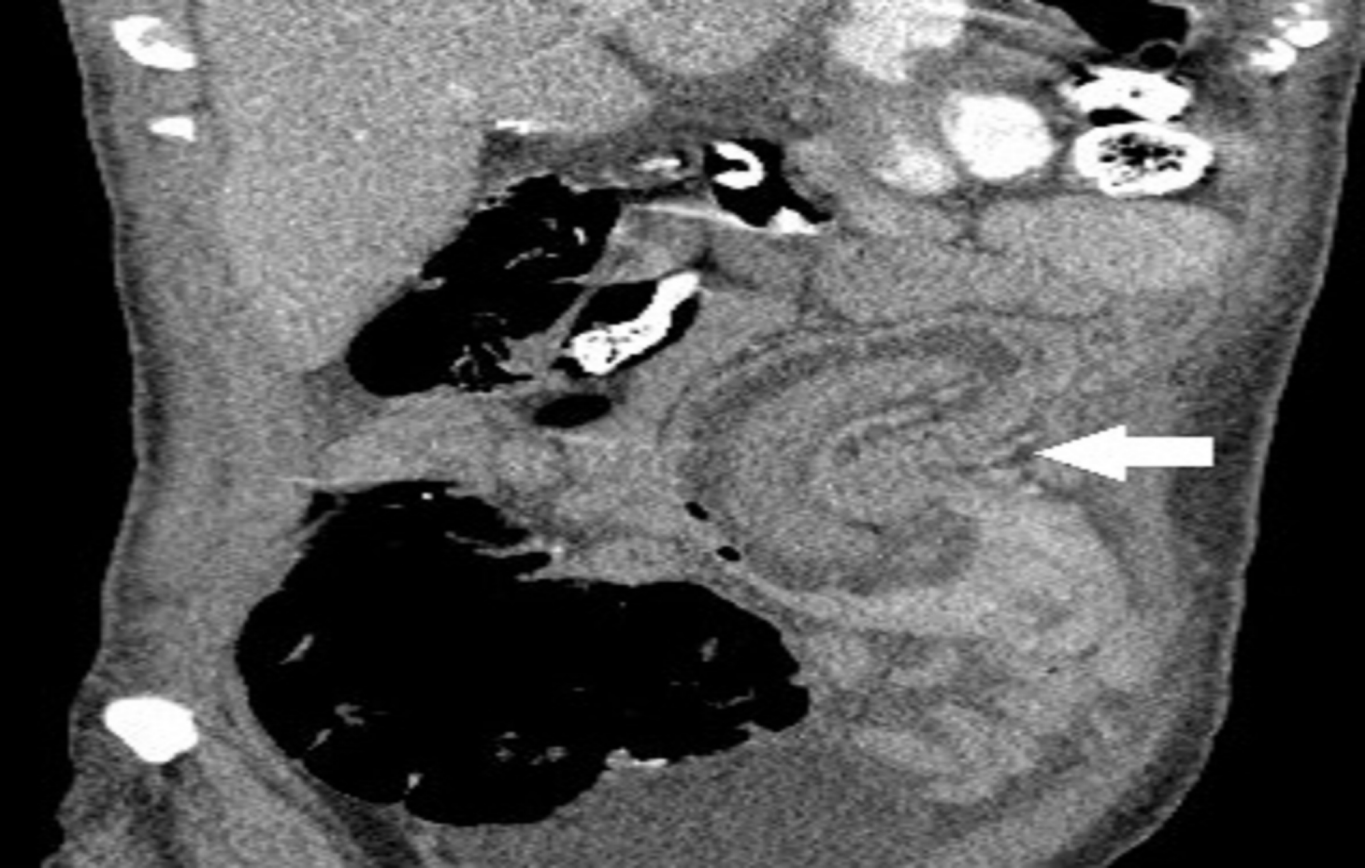
Abdominal computed tomography (CT) scan, coronal view, showing dilatation of the biliopancreatic limb of the REYGB with SB intussusception (arrow) through the jejunojejunostomy REYGB: Roux-en-Y gastric bypass surgery; SB: small bowel.

**Figure 3:**
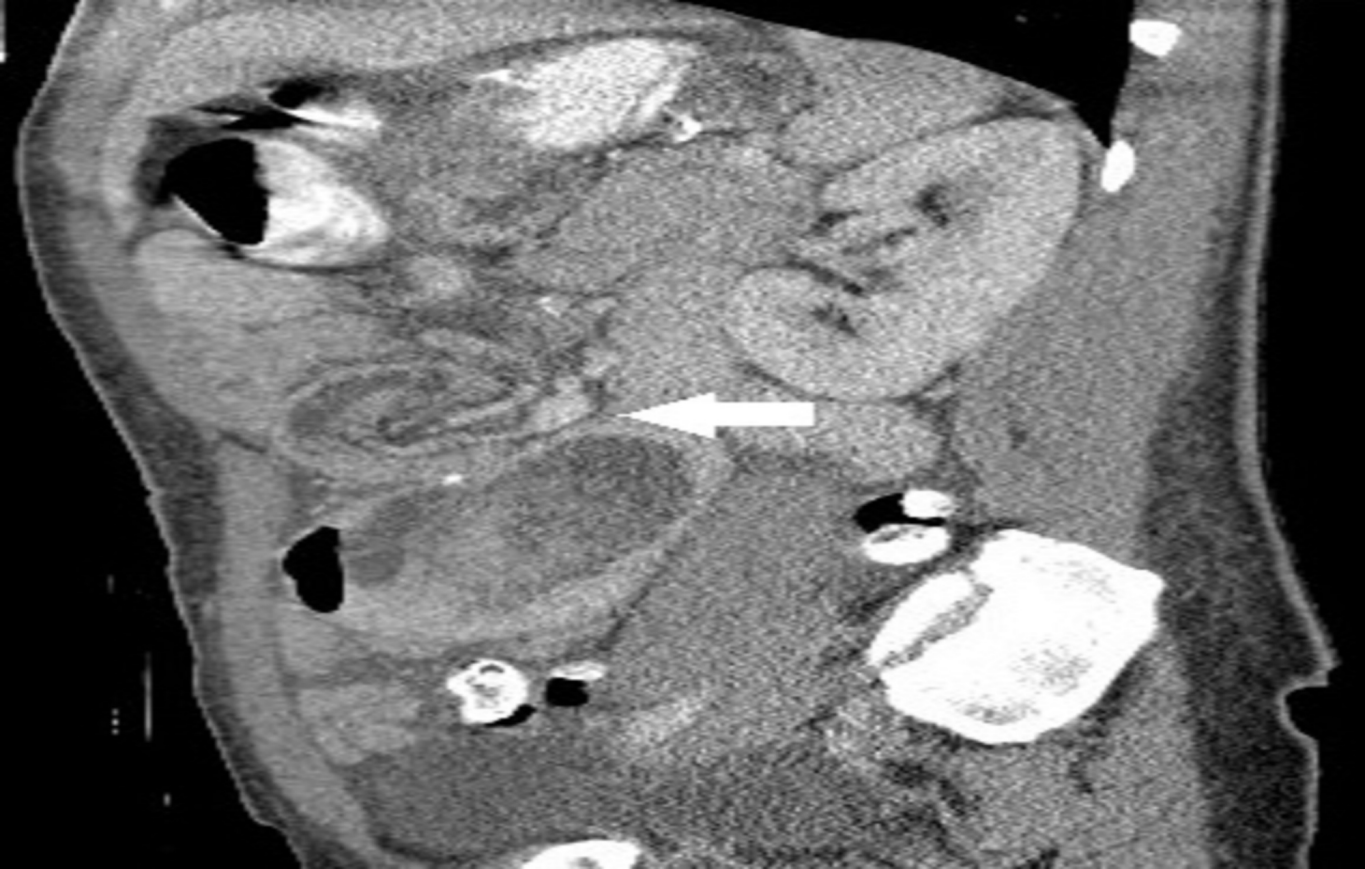
Abdominal computed tomography (CT) scan, sagittal view, showing dilatation of the biliopancreatic limb of the REYGB with SB intussusception (arrow) through the jejunojejunostomy REYGB: Roux-en-Y gastric bypass surgery; SB: small bowel.

A 41-year-old woman who underwent open REYGB six years prior, presented with diffuse crampy abdominal pain, intermittent vomiting, and obstipation of few days. Physical examination revealed abdominal tenderness. Abdominal computed tomography (CT) scan showed dilatation of the biliopancreatic limb of the REYGB with SB intussusception through the jejunojejunostomy (Figures 1-3).

Exploratory laparoscopy was performed with full reduction of a retrograde intussusception where involved bowel loops appeared viable. Extensive adhesions lysis was done and no bowel resection was deemed necessary. Bowel function returned two days after the operation and patient was discharged home.

## Discussion

Intussusception accounts for only 1%-5% of all bowel obstructions in adults. The origin of intussusception after REYGB does not involve a lead point which makes it different from other causes of intussusception. It is suggested that the intussusception in this setting is related to motility disorders in the divided SB which could commonly lead to a retrograde (antiperistaltic) telescoping, as in our patient (Haddad FG, Zaraket V, Deeb L: Retrograde Jejuno-Jejunal Intussusception after Roux-en-Y Gastric Bypass: A Potential à la Mode Complication; Am J Gastroenterol 111: S971-S1076; doi:10.1038/ajg.2016.374). Rarely the telescoping could be anterograde or isoperistaltic [1-3]. This condition may cause obstruction and lead to bowel ischemia and necrosis if not recognized and treated in a timely manner. Symptoms are typically non specific. CT scan represents the diagnostic test of choice, but surgery is the only way to establish the diagnosis in some cases. Management should include early involvement of a bariatric surgeon, and is usually limited to surgical reduction if the SB is still viable. However, resection of the affected segment is still controversial but often recommended, since it seems to result in fewer recurrences [1-4].

## Conclusions

View the hastened blooming of the bariatric era, it is imperative to define the possible complications of bariatric procedures especially on the long term period. Intussusception is a rare but potentially fatal complication that can occur post Roux-en-Y gastric bypass procedure. Increased awareness of this entity and high index of suspicion are required to make the correct diagnosis and offer appropriate treatment in a timely fashion.
